# Defining the Metabolic Pathways and Host-Derived Carbon Substrates Required for Francisella tularensis Intracellular Growth

**DOI:** 10.1128/mBio.01471-18

**Published:** 2018-11-20

**Authors:** Lauren C. Radlinski, Jason Brunton, Shaun Steele, Sharon Taft-Benz, Thomas H. Kawula

**Affiliations:** a Department of Microbiology and Immunology, University of North Carolina at Chapel Hill, Chapel Hill, North Carolina, USA; b Paul G. Allen School for Global Animal Health, Washington State University, Pullman, Washington, USA; University of Chicago

**Keywords:** *Francisella tularensis*, GdhA, GlpA, carbon metabolism, intracellular pathogen

## Abstract

Francisella tularensis is a Gram-negative, facultative, intracellular bacterial pathogen and one of the most virulent organisms known. A hallmark of F. tularensis pathogenesis is the bacterium’s ability to replicate to high densities within the cytoplasm of infected cells in over 250 known host species, including humans. This demonstrates that F. tularensis is adept at modulating its metabolism to fluctuating concentrations of host-derived nutrients. The precise metabolic pathways and nutrients utilized by F. tularensis during intracellular growth, however, are poorly understood. Here, we use systematic mutational analysis to identify the carbon catabolic pathways and host-derived nutrients required for F. tularensis intracellular replication. We demonstrate that the glycolytic enzyme phosphofructokinase (PfkA), and thus glycolysis, is dispensable for F. tularensis SchuS4 virulence, and we highlight the importance of the gluconeogenic enzyme fructose 1,6-bisphosphatase (GlpX). We found that the specific gluconeogenic enzymes that function upstream of GlpX varied based on infection model, indicating that F. tularensis alters its metabolic flux according to the nutrients available within its replicative niche. Despite this flexibility, we found that glutamate dehydrogenase (GdhA) and glycerol 3-phosphate (G3P) dehydrogenase (GlpA) are essential for F. tularensis intracellular replication in all infection models tested. Finally, we demonstrate that host cell lipolysis is required for F. tularensis intracellular proliferation, suggesting that host triglyceride stores represent a primary source of glycerol during intracellular replication. Altogether, the data presented here reveal common nutritional requirements for a bacterium that exhibits characteristic metabolic flexibility during infection.

## Abstract (precis)

The widespread onset of antibiotic resistance prioritizes the need for novel antimicrobial strategies to prevent the spread of disease. With its low infectious dose, broad host range, and high rate of mortality, F. tularensis poses a severe risk to public health and is considered a potential agent for bioterrorism. F. tularensis reaches extreme densities within the host cell cytosol, often replicating 1,000-fold in a single cell within 24 hours. This remarkable rate of growth demonstrates that F. tularensis is adept at harvesting and utilizing host cell nutrients. However, like most intracellular pathogens, the types of nutrients utilized by F. tularensis and how they are acquired is not fully understood. Identifying the essential pathways for F. tularensis replication may reveal new therapeutic strategies for targeting this highly infectious pathogen and may provide insight for improved targeting of intracellular pathogens in general.

## INTRODUCTION

In order to establish a successful infection, intracellular bacterial pathogens must adapt their metabolism to utilize the nutrients available within the host cell, often in direct competition with the host’s own metabolic processes and mechanisms for nutrient sequestration (1). Nevertheless, many of these microorganisms have evolved dedicated mechanisms to harvest and assimilate essential nutrients to proliferate within this specialized niche (2–4). Targeted strategies for carbon acquisition and assimilation fuel bacterial replication and often aid in the evasion of host cell defenses (5–7). Despite their importance, the metabolic pathways and host-derived carbon sources utilized by bacterial pathogens *in vivo* are generally not well understood (8, 9).

Metabolites can be directly acquired from the host, salvaged from similar molecules, or synthesized *de novo* using host-derived sources of carbon, nitrogen, sulfur, etc. Bacteria that replicate within the host cell cytosol theoretically have access to the products and intermediates produced during major host metabolic processes that take place within this compartment, including glycolysis and amino acid biosynthesis. The actual concentrations of these products within an infected cell, however, are unclear. Rather, most nutrients are stored within complex structures, such as lipid droplets, glycogens, and proteins, and thus are not immediately available to intracellular pathogens (8).

Many bacteria employ active mechanisms to acquire host-derived carbon during intracellular growth. Mycobacterium tuberculosis and Chlamydia tracomatis, for instance, associate with host lipid droplets and utilize host-derived lipids for anabolic and catabolic purposes (10, 11). Salmonella enterica serovar Typhimurium secretes effector proteins that stimulate the activation of Akt, a major metabolic regulator of host metabolism (12, 13). This, in turn, stimulates host glycolytic flux and increases the concentration of glucose within the infected cell (13). Similar effector molecules actively alter host vesicular trafficking to direct nutrients to the Salmonella-containing vacuole (14). The observation that many pathogens employ active mechanisms to obtain carbon emphasizes that carbon acquisition within the host cell requires complex host-pathogen interactions, which are only beginning to be elucidated.

We previously demonstrated that Francisella tularensis induces host autophagy during infection, and that this pathway provides the pathogen with essential amino acid metabolites (15). Nevertheless, F. tularensis replicates to a considerable degree in the absence of autophagy, indicating that autophagy-derived nutrients are only a subset of the total required to support full F. tularensis intracellular proliferation (15). A transposon mutagenesis screen of Francisella tularensis subsp. holarctica LVS revealed that nearly half of the genes identified as essential for proliferation in macrophages encode proteins involved in metabolism or metabolite transport (16). These proteins include enzymes predicted to facilitate gluconeogenesis, glycerol catabolism, and amino acid transport, as well as purine, lipopolysaccharide (LPS), and fatty acid biosynthesis. Surprisingly, no glycolytic genes were identified during this screen. Glycolysis is a fundamental metabolic pathway that oxidizes carbohydrates to generate energy and provide precursor metabolites for other biosynthetic pathways. In contrast, the gluconeogenic pathway reverses the reactions of glycolysis during growth on nonglucose carbon substrates to replenish stores of glucose-6-phosphate (glucose-6P) and other essential metabolic intermediates when glucose concentrations are limited. One gene encoding a key gluconeogenic enzyme, *glpX*, was required for efficient intracellular replication (16). Indeed, *glpX* has repeatedly been identified as an important factor for virulence in genetic screens performed in F. tularensis Schu S4 and LVS (17–19). Furthermore, recent work by Brissac et al. demonstrated that gluconeogenesis is an essential metabolic pathway for Francisella novicida and F. tularensis LVS during growth in glucose-limiting conditions (20). These data suggest that F. tularensis intracellular proliferation may not be dependent on glycolysis but rather on gluconeogenesis to preferentially assimilate nonglucose carbon substrates within a host cell.

To determine the specific host-derived carbon sources that facilitate rapid F. tularensis intracellular proliferation, we aimed to define the essential carbon metabolic pathways and metabolites required for F. tularensis intracellular and *in vivo* growth.

## RESULTS

### Gluconeogenesis, but not glycolysis, is essential for F. tularensis intracellular growth and virulence.

**FIG 1:**
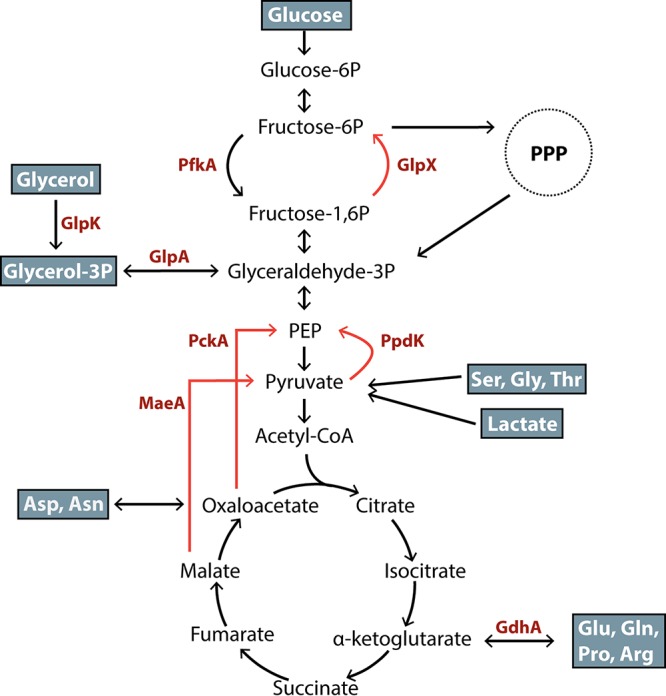
An overview of Francisella tularensis subsp. tularensis Schu S4 central carbon metabolism. Labeled enzymes (red) are predicted to be required for the flux of specific carbon substrates (blue) for F. tularensis central carbon metabolism. Orange arrows indicate reactions that are specific to gluconeogenesis. PPP, pentose phosphate pathway; PEP, phosphoenolpyruvate; acetyl-CoA, acetyl coenzyme A; PfkA, phosphofructokinase (FTT_0801); GlpX, fructose 1,6-bisphosphatase (FTT_1631); GlpK, glycerol kinase (FTT_0130); GlpA, glycerol 3-phosphate dehydrogenase (FTT_0132); PckA, phosphoenolpyruvate carboxykinase (FTT_0449); PpdK, pyruvate phosphate dikinase (FTT_0250); MaeA, malic enzyme (FTT_0917); GdhA, glutamate dehydrogenase (FTT_0380).

Unlike most enzymatic reactions of the glycolytic pathway, the conversion between fructose 6-phosphate (F6P) and fructose 1,6-bisphosphate (FBP) is physiologically irreversible, and is catalyzed by enzymes specific to either glycolysis or gluconeogenesis. In F. tularensis, the glycolytic enzyme phosphofructokinase (PfkA) converts F6P to FBP, and the gluconeogenic enzyme fructose 1,6-bisphosphatase (GlpX) performs the reverse reaction (Fig. 1). Deletion of *pfkA* should prevent F. tularensis from utilizing glucose or glucose 6-phosphate imported from the host, while deletion of *glpX* should prevent the bacterium from producing F6P during growth on gluconeogenic carbon sources. F6P is a precursor of the pentose phosphate pathway and is used for the *de novo* synthesis of lipopolysaccharide, peptidoglycan, pentose phosphates, and aromatic amino acids. We hypothesized that if glucose represents a major carbon source for F. tularensis within the host cell, then *pfkA* would be essential. Alternatively, if glucose is not a major source of carbon utilized by F. tularensis, then the gluconeogenic enzyme *glpX* would be required in order to synthesize sufficient F6P and glucose-6P from alternate carbon sources.

**FIG 2:**
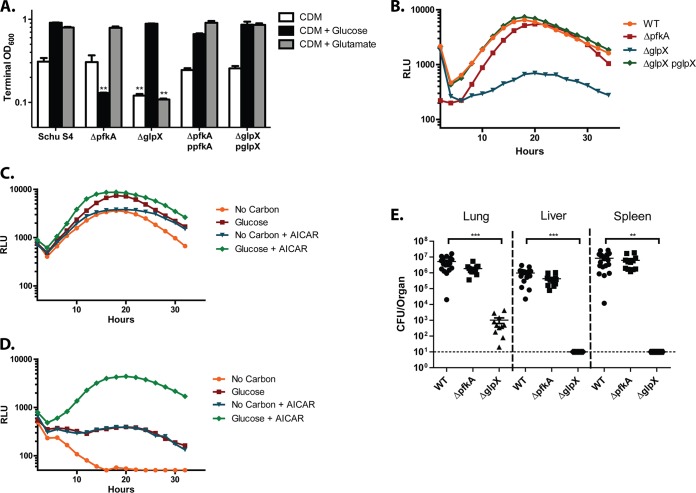
F. tularensis GlpX is essential for replication on gluconeogenic carbon substrates, within host macrophages, and in a murine model of infection. (A) Terminal OD_600_ of WT Schu S4, Δ*pfkA*, and Δ*glpX* strains after 48 h of growth in CDM and CDM supplemented with glucose or glutamate at a final concentration of 0.4%. (B) Intracellular replication of WT Schu S4, Δ*pfkA*, Δ*glpX*, and Δ*glpX* p*glpX* strains in BMDMs, as indicated via relative light units (RLU) measured every 15 min over a 36-hour period. (C) Growth of WT Schu S4 and (D) Δ*glpX* strains in BMDMs cultured with or without 150 µM AICAR and/or glucose at a concentration of 4.5 g/liter. Growth was measured via luminescence read every 15 min over 36 h. Each growth curve represents one of three independent experiments, and each data point represents the average of three technical triplicates. (E) Organ burdens of mice 3 days post intranasal inoculation with WT Schu S4, Δ*pfkA*, and Δ*glpX* strains. Data are pooled from three independent experiments. **, *P* < 0.01, and ***, *P* < 0.001, as determined by Student's *t* test.

We first sought to confirm the predicted functions of *pfkA* and *glpX* for glycolysis and gluconeogenesis, respectively. Markerless, in-frame deletions were created for *pfkA* and *glpX* in Francisella tularensis subsp. tularensis Schu S4, and the deletion strains were grown in defined medium with either glycolytic or gluconeogenic carbon substrates. For all broth cultures, F. tularensis was grown in Chamberlain’s defined medium (CDM) containing a low concentration (∼3mM) of 13 essential and nonessential amino acids and no other major carbon sources (21) (Text S1). In this medium, wild-type (WT) Schu S4 cells grew to low, but detectable levels, presumably by assimilating amino acids for protein synthesis or energy production (Fig. 2A and Fig. S1A). Indeed, Brissac et al. recently demonstrated that supplementation of this medium with 30 mM select amino acids (threonine, proline, methionine, lysine, tyrosine, tryptophan, phenylalanine, asparagine, or serine) permits various degrees of F. novicida growth, suggesting that these amino acids may be utilized as carbon sources (20). Supplementation with either glucose or glutamate supported robust growth of WT Schu S4 cells (Fig. 2A and Fig. S1A). A Δ*pfkA* mutant grew to WT levels during growth on glutamate, but did not grow on glucose, possibly due to glucose-mediated repression of alternative carbon catabolic pathways (Fig. 2A). Importantly, though terminal optical density at 600 nm (OD_600_) describes the overall capacity of each mutant to grow on different carbon substrates, closer attention to *in vitro* doubling times reveals subtle nuances in how each deletion affects growth. For instance, a Δ*pfkA* mutant reaches the same OD_600_ as WT Schu S4, but grows at a much lower rate (Fig. S1B). As expected, the Δ*glpX* mutant grew on glucose but not on glutamate (Fig. 2A and Fig. S1C). The growth defects of each mutant were restored to WT levels when the deleted genes were complemented in *trans* (Fig. 2A and Fig. S1A to C).

10.1128/mBio.01471-18.1 FIG S1 Growth kinetics of F. tularensis Δ*pfkA* and *glpX* mutants during growth in CDM, CDM + glucose and CDM + glutamate. (A) WT Schu S4, (B) Δ*pfkA* and Δ*pfkA* p*pfkA*, and (C) Δ*glpX* and Δ*glpX* p*glpX* strains were grown in CDM and CDM supplemented with glucose or glutamate at a final concentration of 0.4%. The OD_600_ was measured every 15 min over a 36-h period. Each growth curve represents one of three independent experiments, and each data point represents the average of three technical triplicates. Download FIG S1, TIF file, 0.6 MB. Copyright © 2018 Radlinski et al. 2018 Radlinski et al. This content is distributed under the terms of the Creative Commons Attribution 4.0 International license.

10.1128/mBio.01471-18.7 TEXT S1 Supplemental methods. Download Text S1, DOCX file, 0.02 MB. Copyright © 2018 Radlinski et al. 2018 Radlinski et al. This content is distributed under the terms of the Creative Commons Attribution 4.0 International license.

To assess the importance of glycolysis and gluconeogenesis during F. tularensis intracellular growth, we utilized a luminescence reporter to monitor intracellular growth, as previously described (16), where F. tularensis Schu S4 strains harbor a plasmid expressing luciferase enzyme and substrate, as well as an addiction system to maintain the plasmid even in the absence of antibiotic selection. As demonstrated previously, an increase in the bacterial burden within the infected cell is directly proportional to an increase in reporter luminescence (16). Bone marrow-derived macrophages (BMDMs) were infected with WT Schu S4, the Δ*pfkA* mutant, or the Δ*glpX* mutant, each strain harboring the luminescence reporter. Twenty-four hours postinoculation, WT Schu S4 and Δ*pfkA* mutant cells grew to similar levels within the BMDMs, while the growth of the Δ*glpX* mutant was reduced approximately 10-fold relative to that of the WT and the Δ*pfkA* mutant (Fig. 2B). These data indicate that gluconeogenesis, but not glycolysis, is necessary for WT levels of F. tularensis intracellular growth and suggests that glucose does not represent a major carbon source within macrophage cells.

We hypothesized that the severe intracellular growth defect observed for the Δ*glpX* mutant was due to the mutant’s inability to synthesize sufficient levels of F6P and G6P from the catabolism of gluconeogenic carbon substrates. Therefore, supplementation with excess glucose should rescue Δ*glpX* mutant growth within cells. J774A.1 cells are a transformed macrophage cell line constitutively expressing c-Myc, and therefore import large quantities of glucose to increase glycolytic flux (22, 23). To determine if excess glucose could restore the growth defect of the Δ*glpX* mutant, we infected J774A.1 cells with WT Schu S4, the Δ*pfkA* mutant, and the Δ*glpX* mutant, supplied the infected cells with either high-glucose (4.5 g/liter) or glucose-free Dulbecco minimal essential medium (DMEM), and then measured bacterial growth over a 36-hour period. WT Schu S4 and Δ*pfkA* mutant cells exhibited significant growth with or without glucose supplementation (Fig. S2A). As expected, the Δ*glpX* mutant strain did not replicate within J774A.1 cells cultured in glucose-free DMEM; however, intracellular replication was restored to WT levels with excess glucose (Fig. S2B).

10.1128/mBio.01471-18.2 FIG S2 Intracellular growth characteristics of F. tularensis Schu S4 and mutant strains in J774A.1 macrophage cells. Representative intracellular bacterial growth kinetics of (A) Δ*pfkA*, (B) Δ*glpX* and Δ*glpX* p*glpX*, (C) Δ*pckA*, (D) Δ*ppdK* and Δ*ppdK* p*ppdK.* (E) Δ*gdhA* and Δ*gdhA* p*gdhA*, and (F) Δ*glpKA* and Δ*glpKA* p*glpAF* deletion strains carrying a luciferase (Lux) reporter for intracellular growth within J774A.1 macrophage cells cultured with and without glucose. Luminescence was measured every 15 minutes over a 36-h period, and each point represents the average of three technical replicates. Each panel is a representative of at least 2 independent experiments. Download FIG S2, TIF file, 1.6 MB. Copyright © 2018 Radlinski et al. 2018 Radlinski et al. This content is distributed under the terms of the Creative Commons Attribution 4.0 International license.

The rescue of the Δ*glpX* mutant did not occur in primary BMDMs, as all BMDM infections were performed in high-glucose (4.5g/liter) DMEM (Fig. 2B). This observation suggests that the reduced level of glucose import and glycolytic flux exhibited by BMDMs, relative to those of J774A.1 cells, is insufficient to permit Δ*glpX* cells from acquiring adequate glucose from the host to restore WT growth properties, even when glucose is present at high concentrations in the medium. To test this, we attempted to rescue growth of Δ*glpX* in BMDMs by treating the host cells with 5-amnoimidazole-4-carboxamide ribonucleotide (AICAR). AICAR is an analog of AMP (AMP) that stimulates activation of the major host metabolic regulator, AMP-dependent protein kinase (AMPK) (24). When activated, AMPK stimulates glucose uptake and energy production in part by increasing expression of major glucose transporters GLUT1 and GLUT4, and by increasing overall host glycolytic flux (25). We hypothesized that AICAR treatment of BMDMs cultured in high-glucose DMEM would restore Δ*glpX* mutant intracellular growth by stimulating glucose import. Indeed, while AICAR had little impact on the growth of WT Schu S4 within BMDMs (Fig. 2C), AICAR treatment significantly increased the intracellular growth of Δ*glpX* in BMDMs cultured in high-glucose DMEM (Fig. 2D). Altogether, our results support the conclusion that the inability of the Δ*glpX* mutant to fully assimilate gluconeogenic carbon sources results in attenuated growth during periods of glucose limitation.

We next tested whether F. tularensis similarly requires *glpX* and not *pfkA* for replication in a murine model of F. tularensis pulmonary infection. Groups of C57BL6/J female mice were infected intranasally with 100 CFU of WT, Δ*pfkA*, or Δ*glpX* Schu S4 strains. Three days postinfection, the lungs, livers, and spleens of the infected mice were harvested, homogenized, and plated for bacterial enumeration. Organ burdens for the Δ*pfkA* mutant strain were similar to those of WT Schu S4 (Fig. 2E). However, the number of CFU recovered from the lungs of mice infected with the Δ*glpX* mutant was similar to that of the original inoculum and below the limit of detection in the liver and spleen (Fig. 2E). These data align with our observations that *glpX*, and therefore gluconeogenesis, is necessary for F. tularensis replication in host cells, whereas *pfkA* and glycolysis are dispensable.

### F. tularensis possesses multiple pathways that supply gluconeogenic substrates to support intracellular growth.

Our data suggest that a Δ*glpX* mutant does not produce essential biosynthetic precursors from the nutrients available within the host cell. Since the deletion of *glpX* precludes the utilization of a large number of gluconeogenic carbon sources, such as glycerol, pentose sugars, amino acids, lactate, pyruvate, and tricarboxylic acid (TCA) cycle intermediates, we generated F. tularensis mutant strains unable to utilize some of these specific carbon sources. Like the conversion of F6P to FBP, the enzymatic conversion of pyruvate to phosphoenol pyruvate (PEP) during glycolysis is physiologically irreversible and must be bypassed during gluconeogenesis. The ATP-dependent decarboxylation of oxaloacetate to PEP is catalyzed by PEP carboxykinase (*pckA*) and is important for growth on carboxylic or amino acids. Alternatively, pyruvate-phosphate dikinase (*ppdK*) converts pyruvate to PEP and is required for growth on pyruvate, lactate, and some amino acids. The reactions catalyzed by each enzyme can independently fuel the gluconeogenic pathway to generate essential metabolic precursors necessary for growth. (Fig. 1).

**FIG 3:**
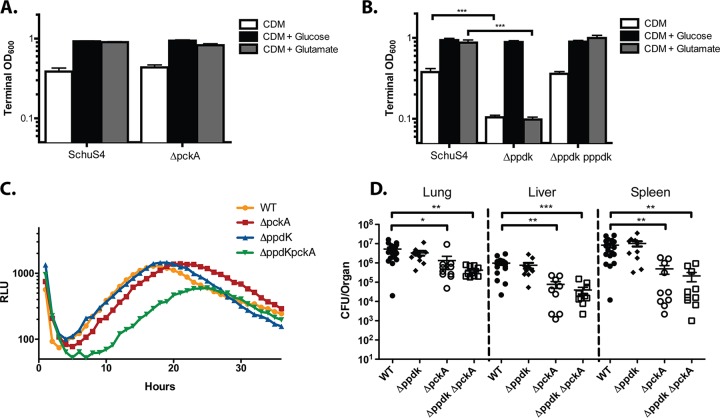
Growth of Δ*pckA* and Δ*ppdK* mutants in defined medium, host cells, and a murine model of infection. Terminal OD_600_ of (A) Δ*pckA* and (B) Δ*ppdK* and Δ*ppdK* p*ppdK* strains after 48 h of growth in CDM and CDM supplemented with glucose or glutamate at a final concentration of 0.4%. Data are pooled from three triplicate wells from three independent experiments (mean ± standard deviation [SD]). (C) The intracellular growth kinetics of Δ*pckA*, Δ*ppdK*, and Δ*ppdK* Δ*pckA* mutants cultured in high-glucose (4.5 g/liter) DMEM, as indicated via RLU measured every 15 min over a 36-hour period. The data shown represent three independent experiments, and each data point represents the average of three technical replicates. (D) Organ burdens of mice 3 days post intranasal inoculation with WT Schu S4, Δ*ppdK*, Δ*pckA*, or Δ*ppdK* Δ*pckA* mutants. Data are pooled from three independent experiments. *, *P* < 0.05; **, *P* < 0.01; and ***, *P* < 0.001, as determined by Student's *t* test.

We generated markerless, in-frame deletions of *ppdK* and *pckA*. Growth characteristics of each mutant were analyzed in defined medium with specific glycolytic or gluconeogenic substrates to confirm the metabolic function of each enzyme. Both Δ*ppdK* and Δ*pckA* mutants grew to levels similar to that of WT Schu S4 in CDM supplemented with glucose (Fig. 3A and B). However, while the Δ*pckA* mutant grew to WT levels in CDM with or without excess glutamate, the Δ*ppdK* mutant had a severe growth defect in CDM and in CDM supplemented with glutamate, similar to that of the Δ*glpX* mutant (Fig. 3A and B). These data suggest that during growth in defined medium, F. tularensis preferentially synthesizes PEP from pyruvate (PpdK) and not from oxaloacetate (PckA).

We observed that both the Δ*ppdK* and Δ*pckA* mutants grew to WT levels within the BMDMs (Fig. 3C). Furthermore, a Δ*ppdK* Δ*pckA* double mutant replicated to significant levels within these cells, albeit at a lower rate, suggesting that these gluconeogenic pathways are not essential for F. tularensis growth within BMDMs (Fig. 3C). When we infected J774A.1 cells with the Δ*ppdK* and Δ*pckA* mutants, we found that the Δ*pckA* mutant grew to WT levels within J774A.1 cells cultured with or without glucose supplementation (Fig. S2C). The Δ*ppdK* mutant, however, exhibited significantly reduced growth within J774A.1 cells cultured without glucose (Fig. S2D). Intracellular proliferation of the Δ*ppdK* mutant was restored to WT levels upon high glucose supplementation, indicating that *ppdK* may contribute to the assimilation of host-derived carbon in J774A.1 cells.

While we found that the organ burdens of the Δ*ppdK* mutant were similar to those of WT Schu S4 in our murine model, we recovered significantly reduced numbers of the Δ*pckA* mutant from the lungs, livers, and spleens of infected mice (Fig. 3D). Furthermore, we recovered similar numbers of the Δ*ppdK* Δ*pckA* double mutant relative to that of the Δ*pckA* single mutant. This indicates that *pckA* is required for optimal replication in a murine model of F. tularensis infection but that *ppdK* is dispensable (Fig. 3D).

### Amino acids feed the gluconeogenic pathway through the TCA cycle.

The attenuation of the Δ*pckA* mutant in mice suggests that F. tularensis relies on the metabolic pathway catalyzed by PckA during infection. Potential nutrients that can fuel the gluconeogenic pathway through PckA include TCA cycle intermediates or amino acids that feed into the TCA cycle. To discern between these possibilities, we evaluated the importance of glutamate dehydrogenase (*gdhA*) for F. tularensis intracellular growth. GdhA catalyzes the reversible oxidative deamination of glutamate to α-ketoglutarate, a TCA cycle intermediate (Fig. 1). F. tularensis is predicted to require GdhA to shuttle several amino acids into the TCA cycle, including glutamate, glutamine, proline, arginine, and potentially aspartate and asparagine. Therefore, if F. tularensis preferentially catabolizes amino acids and not TCA cycle intermediates, then a Δ*gdhA* mutant would likely be similarly attenuated relative to Δ*pckA* during *in vivo* growth.

**FIG 4:**
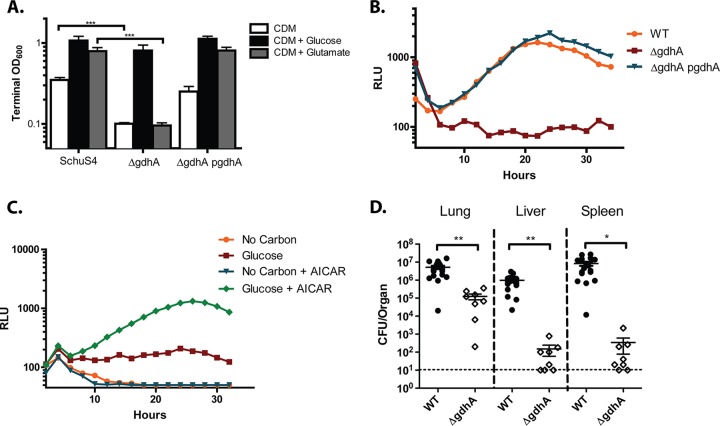
GdhA fuels gluconeogenesis by shuttling carbon into the TCA cycle. (A) Terminal OD_600_ of Δ*gdhA* cells after 48 h of growth in CDM and CDM supplemented with glucose or glutamate at a final concentration of 0.4%. Data are pooled from three triplicate wells from three independent experiments (mean ± SD). (B) The intracellular growth kinetics of WT Schu S4, Δ*gdhA*, and Δ*gdhA* p*gdhA* strains within BMDMs, as indicated via RLU measured every 15 min over a 36-h period. (C) Δ*gdhA* strains expressing the luciferase (Lux) reporter of intracellular growth in BMDMs cultured with or without 150 µM AICAR and/or glucose at a concentration of 4.5 g/liter. Each growth curve represents one of three independent experiments, and each data point represents the average of three technical triplicates. (D) Organ burdens of mice 3 days post intranasal inoculation with WT Schu S4 or the Δ*gdhA* mutant. Data are pooled from three independent experiments. *, *P* < 0.05; **, *P* < 0.01; and ***, *P* < 0.001, as determined by Student's *t* test.

To validate the predicted function of *gdhA*, we tested a Δ*gdhA* mutant for growth on glycolytic and gluconeogenic carbon sources in defined medium. As expected, we found that *gdhA* was required for growth in CDM or CDM supplemented with glutamate, but not in CDM supplemented with glucose (Fig. 4A). Because the Δ*gdhA* mutant grew to significant levels on glucose in defined media lacking glutamate, we reasoned that *gdhA* was dispensable for glutamate synthesis but was required for glutamate assimilation.

The Δ*gdhA* deletion mutant exhibited reduced growth in BMDMs that was restored upon expression of *gdhA* in *trans* (Fig. 4B), suggesting that GdhA-mediated carbon assimilation represents an important metabolic pathway during F. tularensis replication in BMDMs. When we infected J774A.1 macrophage cells with the Δ*gdhA* mutant, we found that the defect in bacterial intracellular replication during culture in glucose-free DMEM could be partially rescued with excess glucose, similar to that of the Δ*glpX* and Δ*ppdK* mutants (Fig. S2E). Similarly, BMDMs cultured in high-glucose DMEM and treated with AICAR permitted significant growth of the Δ*gdhA* mutant relative to that in untreated BMDMs (Fig. 4C). These data suggest that the intracellular growth defect observed for the Δ*gdhA* mutant is at least in part due to the ability of this mutant to assimilate sufficient host-derived carbon.

We expected the Δ*gdhA* mutant to be similarly attenuated relative to a Δ*pckA* mutant during growth in mice. Strikingly, when we assessed the requirement of *gdhA* for growth in our murine model of *F. tularensis* pulmonary infection, we found that the CFU recovered from the lungs, livers, and spleens of *gdhA-*infected mice were greatly reduced compared to those from the WT, and that the CFU recovered from the liver and spleen of the mice were reduced approximately 3-fold relative to those from the Δ*pckA* mutant (Fig. 4D). In addition to fueling the gluconeogenic pathway, Δ*gdhA-*mediated anaplerosis of the TCA cycle may be essential during infection to supply other essential metabolic precursors (e.g., oxaloacetate and/or acetyl coenzyme A [acetyl-CoA]) or to generate reducing power via the use of malic enzyme. This conclusion is consistent with our observation that excess glucose supplementation during growth in J774A.1 or AICAR-treated BMDMs only partially rescued bacterial proliferation of this mutant.

### Glycerol catabolism is required for F. tularensis *in vivo* growth.

Since the Δ*glpX* mutant was more severely attenuated in mice relative to the Δ*ppdK* Δ*pckA* mutant, we reasoned that F. tularensis may assimilate additional carbon substrates besides those supplied through the gluconeogenic pathways catalyzed by Ppdk and PckA. In F. tularensis, glpA (G3P dehydrogenase) is predicted to be required for the catabolism of glycerol and G3P. We used the Targetron gene knockout system modified for use in Francisella to disrupt *glpA* in F. tularensis Schu S4 (26). Interestingly, we found that the generation of a Δ*glpA* mutant strain was only possible through the simultaneous introduction of a secondary mutation in *glpK* (Fig. S3A). In F. tularensis, *glpK* is located upstream of *glpA* and is predicted to encode a kinase responsible for the phosphorylation of glycerol forming G3P during glycerol catabolism (Fig. S3A). The disruption of G3P dehydrogenase in the presence of a fully functional glycerol kinase can lead to increased concentration of intracellular G3P. In Escherichia coli, excess G3P within the cell stimulates the synthesis of the toxic metabolite methylglyoxal (27). We suspect that a similar phenomenon may be responsible for the requirement of a secondary *glpK* mutation in an F. tularensis Δ*glpA* background.

10.1128/mBio.01471-18.3 FIG S3 GlpA, but not GlpK, is required for growth on glycerol 3-phosphate (G3P) and in BMDMs. (A) Schematic representing the coding regions for *glpK*, *glpA,* and *glpF*, including the location of the missense substitution mutation (resulting in an arginine to cysteine substitution) introduced at position 1084 in *glpK* during the generation of the *glpA* insertion mutant. *(glpK*, glycerol kinase; *isftu1*, insertion sequence element; *glpA*, G3P dehydrogenase; *glpF*, glycerol uptake facilitator). (B) Terminal OD_600_ of WT Schu S4, the *glpKA* insertion mutant, and the corresponding p*glpAF* complemented strain grown in CDM and CDM supplemented with glucose, glycerol, or G3P after 48 h of growth. (C) Terminal RLU values for BMDMs infected with WT Schu S4, Δ*glpKA*, or Δ*glpKA* p*glpAF* strains harboring a LUX reporter for intracellular growth. The panel is representative of two independent experiments, and data shown are averages from three triplicate wells (mean ± SD). Data are pooled from three triplicate wells from three independent experiments (mean ± SD). Download FIG S3, TIF file, 1.0 MB. Copyright © 2018 Radlinski et al. 2018 Radlinski et al. This content is distributed under the terms of the Creative Commons Attribution 4.0 International license.

**FIG 5:**
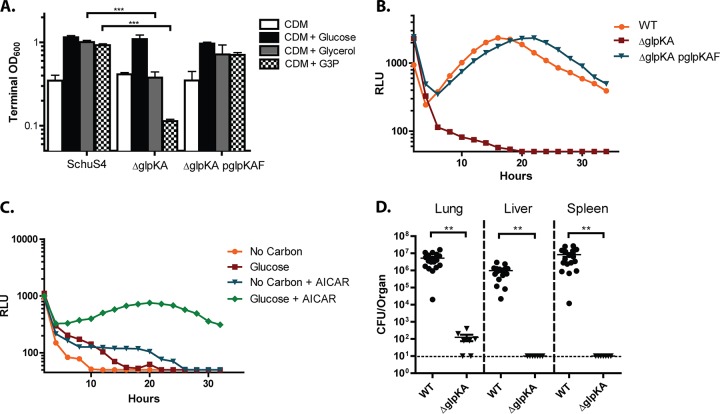
Glycerol metabolism is essential for F. tularensis intracellular replication. (A) Terminal OD_600_ of WT Schu S4, the *glpKA* insertion mutant, and the corresponding p*glpKAF* complemented strain grown in CDM and CDM supplemented with glucose, glycerol, or G3P after 48 h of growth. Data are pooled from three triplicate wells from three independent experiments (mean ± SD). (B) Growth curves of WT Schu S4, the *glpKA* mutant, and the *glpKAF* complemented strain harboring the LUX reporter within BMDMs. Intracellular bacterial growth was measured via luminescence (RLU), read every 15 min over a 36-hour period. (C) Growth of Δ*glpA* mutant expressing the LUX reporter of intracellular growth in BMDMs cultured with or without 150 µM AICAR and/or glucose at a concentration of 4.5 g/liter. Each growth curve represents one of three independent experiments, and each data point represents the average of three technical triplicates. (D) Organ burdens of mice 3 days post intranasal inoculation with WT Schu S4 and the *glpKA* insertional mutant. Data are pooled from three independent experiments. *, *P* < 0.05; **, *P* < 0.01; and ***, *P* < 0.001, as determined by Student's *t* test.

We analyzed the growth properties of the *glpKA* disruption mutant in defined medium supplemented with glucose, glycerol, or G3P to confirm that *glpA* and *glpK* are required for growth on glycerol and G3P. As expected, the *glpKA* mutant grew to WT levels when cultured with glucose but not with glycerol or G3P (Fig. 5A). In fact, supplying the *glpKA* mutant with G3P led to significantly lower levels of bacterial replication relative to growth on glycerol or just CDM, possibly due to the toxic buildup of intracellular G3P. We found that growth of the *glpKA* mutant was restored on glycerol and G3P only when these two genes were expressed with the downstream gene, *glpF*, despite the fact that sequencing of the surrounding genes in our *glpKA* mutant revealed no additional mutations in or around the coding sequence for *glpF. glpF* is predicted to encode a glycerol uptake facilitator and may be cotranscribed with *glpA* (Fig. S3A). As expected, we observed WT levels of growth on G3P but not on glycerol when *glpA* and *glpF*, but not *glpK*, were expressed in *trans* in the *glpKA* mutant (Fig. S3B), and growth on both glycerol and G3P was restored when the *glpKA* mutant was complemented with *glpKAF* in *trans* (Fig. 5A). These findings are summarized in Table S1.

10.1128/mBio.01471-18.5 TABLE S1 Summary of Schu S4 and *glpKA* strain growth in broth, BMDMs, or J774A.1 cells. Data are summarized from Fig. 5 in the main text and Fig. S3 in the supplemental material. –, no growth; +, intermediate levels of growth; ++, wild-type levels of growth; n/a, not tested; CDM, Chamberlain’s defined medium; G3P, glycerol 3-phosphate; BMDM, bone marrow-derived macrophage. Download Table S1, DOCX file, 0.01 MB. Copyright © 2018 Radlinski et al. 2018 Radlinski et al. This content is distributed under the terms of the Creative Commons Attribution 4.0 International license.

The *glpKA* mutant replicated in J774A.1 cells to intermediate levels with or without supplemented glucose, indicating that this mutant can replicate within this cell line, but growth was not fully restored by the addition of excess glucose (Fig. S2F). We found that the *glpKA* disruption mutant did not replicate within BMDMs (Fig. 5B). Interestingly, growth within BMDMs was restored to WT levels upon in *trans* expression of *glpA* and *glpF* without *glpK,* suggesting that F. tularensis Schu S4 may assimilate G3P and not glycerol during intracellular growth (Fig. S3C). Growth of the mutant within BMDMs was similarly restored to WT levels upon complementation of *glpKAF* (Fig. 5B). Finally, replication of the *glpKA* mutant was significantly increased within BMDMs cultured with AICAR and excess glucose, demonstrating that, similar to the *glpX* mutant, the *glpKA* disruption mutant could be rescued by supplying an alternative carbon source (Fig. 5C).

We then assessed the importance of glycerol catabolism for F. tularensis during growth in mice and found that the number of CFU recovered from the lungs of mice infected with the *glpKA* disruption mutant was similar to the original inoculum and below the limit of detection in the livers and spleens of mice (Fig. 5D). These data suggest that a *glpKA* mutant colonized, but did not proliferate or disseminate, in a murine model of F. tularensis infection.

**FIG 6:**
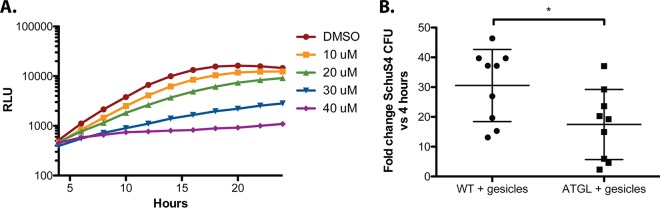
Active host cell lipolysis is required for efficient F. tularensis intracellular replication. (A) Growth curve of WT Schu S4 cells harboring a LUX reporter within BMDMs cultured in high-glucose (4.5 g/liter) DMEM with or without Atglistatin at indicated concentrations. Intracellular bacterial growth was measured via luminescence (RLU), read every 15 min over a 24-h period. Data represent the mean pooled from 3 replicates in 3 independent experiments. (B) Fold change in WT Schu S4 burden between 24 and 4 h postinfection of WT and ATGL knockdown BMDMs. Data are pooled from three independent experiments. *, *P* < 0.05; **, *P* < 0.01; and ***, *P* < 0.001, as determined by Student's *t* test).

Data from our mutational analysis suggest that glycerol represents an essential host-derived source of carbon during F. tularensis intracellular growth. However, we could not exclude the alternative possibility that F. tularensis attenuation may be due to a toxic buildup of metabolites or disruption of proper metabolic regulatory mechanisms in our mutant strains. To delineate these possibilities, we sought to reduce the concentration of available glycerol within BMDMs and to examine the impact on WT F. tularensis intracellular proliferation. A significant bulk of host glycerol stores are sequestered as triglycerides in host lipid droplets (28). During lipolysis, a series of enzymatic reactions free glycerol from cellular triglyceride stores and release it into the cytosol of the host (28, 29). We hypothesized that F. tularensis may exploit this process to establish a source of glycerol during intracellular growth. Atglistatin is a selective inhibitor of adipose triglyceride lipase (ATGL), an enzyme responsible for the first catalytic step of lipolysis (30). When we infected Atglistatin-treated BMDMs with WT F. tularensis, we observed that Atglistatin treatment significantly reduced F. tularensis intracellular burden in a dose-dependent manner (Fig. 6A). Importantly, these concentrations were not cytotoxic to BMDMs (Fig. S4A). To verify these findings, we used Cre-Lox recombination to generate ATGL deficient BMDMs. BMDMs derived from C57Bl6/J or ATGL-flox mice were treated with Cre recombinase gesicles during differentiation. Cre-treated BMDMs isolated from ATGL-flox mice demonstrated approximately 60% knockdown of ATGL expression based on reverse transcription-quantitative PCR (qRT-PCR) (Fig. S4B). This was associated with a significant reduction in F. tularensis replication within ATGL knockdown BMDMs (Fig. 6B). From these data, we conclude that host lipolysis is important for sustaining F. tularensis growth, and that host-derived glycerol represents a primary source of carbon necessary for fueling F. tularensis *in vivo* replication.

10.1128/mBio.01471-18.4 FIG S4 ATGL inhibition reduces F. tularensis growth within BMDMs without cytotoxicity that can be partially rescued by exogenous glycerol. (A) BMDMs were treated with various concentrations of Atglistatin or vehicle control and allowed to incubate for 36 h before BMDM cytotoxicity was measured using a Vybrant MTT [3-(4,5-dimethylthiazol-2-yl)-2,5-diphenyltetrazolium bromide] assay. Data are pooled from three triplicate wells from three independent experiments (mean ± SD). (B) BMDMs were treated with 30 μM Atglistatin or vehicle control, then infected with F. tularensis Schu S4 in the presence or absence of the indicated concentrations of exogenous glycerol. Bacterial replication was monitored via change in luminescence. (C) Quantification of F. tularensis intracellular replication in Atglistatin-treated and untreated BMDMs ± exogenous glycerol, normalized to replication in cells without glycerol. (D) Reverse transcription-quantitative PCR (qRT-PCR) quantification of ATGL expression in WT or ATGL knockdown BMDMs in the presence or absence of Cre recombinase-containing gesicles. Data are represented as fold change relative to the wild type. Data from panels A and D are pooled from three triplicate wells from three independent experiments (mean ± SD). Panels B and C are representative of three independent experiments (*, *P* < 0.01 by Student’s *t* test). Download FIG S4, TIF file, 1.3 MB. Copyright © 2018 Radlinski et al. 2018 Radlinski et al. This content is distributed under the terms of the Creative Commons Attribution 4.0 International license.

## DISCUSSION

Previous work by our group and others highlight the importance of amino acid metabolism for F. tularensis replication and virulence (15, 31–33). Furthermore, Brissac et al. recently demonstrated that gluconeogenesis is vital for F. tularensis subsp. holarctica LVS and F. novicida growth during periods of glucose limitation (20). Here, we have similarly demonstrated that gluconeogenesis is essential for intracellular and *in vivo* growth for the highly virulent F. tularensis subsp. tularensis Schu S4, while *pfkA,* and thus glycolysis, is dispensable. Additionally, through systematic mutational analysis, we identified specific metabolic pathways essential for F. tularensis virulence. We found that Δ*glpX,* Δ*pckA,* Δ*gdhA*, and Δ*glpA* mutant strains were attenuated during growth in a mouse model of F. tularensis pulmonary infection, suggesting that these pathways may by critical for the efficient assimilation of host-derived carbon. These findings are summarized in Table S2.

10.1128/mBio.01471-18.6 TABLE S2 Summary table of Schu S4 WT and mutant strain growth in defined media and infection models. Data are summarized from Fig. S2 and Fig. 5 in the main text. –, no growth; +, intermediate levels of growth; ++, wild type levels of growth; n/a, not tested; CDM, Chamberlain’s defined medium; BMDM, bone marrow-derived macrophage. Download Table S2, DOCX file, 0.01 MB. Copyright © 2018 Radlinski et al. 2018 Radlinski et al. This content is distributed under the terms of the Creative Commons Attribution 4.0 International license.

The metabolic pathways required for F. tularensis growth varied based on the infection model. We found that *pckA* was important for growth in mice, while *ppdK* was essential for WT levels of growth within a J774A.1 transformed macrophage cell line. The differential requirements of these genes suggest that F. tularensis may utilize alternate gluconeogenic pathways for growth in different environments, as the bacterium may preferentially assimilate different host-derived carbon sources, perhaps based on availability. As transformed macrophages undergo altered metabolism relative to primary cells, it is likely that the carbon sources available to F. tularensis are distinct within these models. For instance, J774A.1 metabolism is subject to the “Warburg effect,” in which these cells significantly increase glucose uptake and aerobic glycolysis, leading to high intracellular concentrations of lactate (34). F. tularensis may exploit this metabolic aberrance and primarily assimilate lactate during replication within these cells. As *ppdK* is required for F. tularensis assimilation of lactate (Fig. 1), this may explain the requirement of *ppdK* specifically in J774A.1 cells.

We found that *ppdK*, and not *pckA*, is essential for growth on glutamate in defined medium. F. tularensis possesses an additional gluconeogenic enzyme (malate dehydrogenase [MaeA]) responsible for the synthesis of pyruvate from malate, which can then be converted to PEP through PpdK (Fig. 1). Previous work has suggested little or no utilization of the oxidative branch of the pentose phosphate pathway during F. tularensis growth (20). Bypassing the oxidative branch of the pentose phosphate pathway means that F. tularensis must use an alternative mechanism for the generation of the essential cofactor, NADPH. It is possible that the bacterium relies on an NADP^+^-dependent malic enzyme for the production of NADPH during growth on glutamate defined medium. As the conversion of TCA intermediates to PEP through malic enzyme bypasses PckA but requires PpdK, this would provide a possible explanation for why *ppdK* and not *pckA* is the preferred gluconeogenic pathway during growth on glutamate.

We were surprised to find that a Δ*gdhA* mutant demonstrated significantly reduced growth within a mouse compared to a Δ*ppdK* Δ*pckA* double mutant. If *gdhA* is required solely for gluconeogenic purposes, we would expect that these two mutants would be similarly attenuated, as a *ΔppdK* Δ*pckA* double mutant theoretically halts the gluconeogenic conversion of TCA cycle intermediates to glucose. However, during replication within a mouse, *gdhA* may be additionally required for anaplerosis of the TCA cycle or glutamate biosynthesis. Furthermore, it was recently demonstrated that glutamate import plays a critical role in oxidative stress defense and phagosomal escape during F. tularensis infection (32). Thus, the attenuation of this mutant may be in part due to its inability to withstand oxidative stress within the phagosome to reach the host cell cytosol. However, because growth of Δ*gdhA* can be partially rescued by supplying J774A.1 cells (Fig. S2E) or AICAR-treated BMDMs (Fig. 4C) with excess glucose, we conclude that this pathway is primarily involved in carbon acquisition during F. tularensis intracellular growth.

Unlike *ppdK* and *pckA,* we found that a *glpKA* mutant was attenuated for growth in all models tested, highlighting the importance of glycerol catabolism for F. tularensis pathogenesis. Based on the annotated genomic sequence of F. tularensis subsp. tularensis Schu S4, a Δ*glpA* mutant strain cannot assimilate glycerol or G3P (35). During our investigation, we found that disrupting *glpA* in F. tularensis Schu S4 resulted in an independent polar mutation in *glpK* that prevented growth on glycerol. As expected, genetic complementation of our *glpA* mutant strain with *glpA*, but not with *glpK*, rescued growth on G3P but not on glycerol (Fig. S3C). However, our partially complemented strain replicated to WT levels within BMDMs, suggesting that within this cell type, G3P and not glycerol is available for F. tularensis metabolism. This conclusion is consistent with the fact that glycerol is actively phosphorylated by the host to prevent its efflux from the cell. Of note, unlike F. tularensis subsp. tularensis and F. novicida, F. tularensis subsp. holarctica can only metabolize G3P and not glycerol. As F. tularensis possesses a small, decaying genome adapted to an intracellular lifestyle, this may reflect an interesting evolutionary example that supports our prediction that F. tularensis specifically metabolizes G3P within the cell (36).

Despite occupying similar niches, intracellular bacterial pathogens have evolved distinct methods to meet their respective nutritional requirements. Many pathogens, such as Salmonella enterica, Legionella pneumophila, and enteroinvasive Escherichia coli species, preferentially assimilate glucose during intracellular growth (5, 37, 38). In contrast, Shigella flexneri downregulates genes involved in glucose catabolism and favors the assimilation of C_3_ substrates during growth within the cytosol (39). Listeria monocytogenes relies on two major carbon substrates (glycerol and glucose 6-phosphate) to fuel distinct catabolic and anabolic pathways during cytosolic replication (40). Our data suggest that the primary carbon substrate utilized by F. tularensis during intracellular growth varies depending on the model of infection. This is not surprising, considering that the host range of F. tularensis subsp. tularensis Schu S4 includes over 250 species, and that within these hosts, F. tularensis infects numerous cell types, including macrophages and dendritic, endothelial and epithelial cells (41). In order to replicate within such a diverse range of hosts, F. tularensis must adapt its metabolism to the carbon sources available from the environment, which can vary significantly from host to host and between cell types. Thus, we suspect that the extraordinary ability of F. tularensis to proliferate within such a wide range of hosts is in part due to the pathogen’s capability to sense and adapt to the fluctuating availability of nutrients over the course of its infectious lifestyle.

When available, F. tularensis will consume glucose. The intracellular growth defect of the Δ*glpX* mutant in J774A.1 cells was rescued by supplying excess glucose (Fig. 3B). Furthermore, F. tularensis subsp. holarctica LVS replication within J774A.1 and THP-1 macrophage cells leads to a significant reduction in host intracellular glucose (20). However, the nutrient concentrations within these established cell lines do not reflect the physiological conditions encountered by F. tularensis during infection, and it is likely that in physiological conditions, glucose limitation forces F. tularensis to utilize nonglucose carbon substrates. Indeed, a transcriptomic analysis of the F. tularensis metabolic network during extracellular and intracellular growth suggests that significant changes in carbohydrate metabolism occur when the pathogen transitions to an intracellular lifestyle (42). Our data support the proposed model that in the absence of glucose, F. tularensis will primarily utilize alternate carbon sources, such as amino acids or C_3_ substrates derived from the host.

Bacterial metabolic pathways must be coordinated to reduce unnecessary energy expenditure and maximize fitness. In E. coli, key branch points in the glycolytic pathway are controlled by feed-forward/feedback inhibition. For instance, the conversion of F6P to FBP by PfkA is stimulated by ADP and inhibited by the downstream metabolite PEP (43). Conversely, the reverse reaction (catalyzed by fructose 1,6-bisphosphatase) is inhibited by AMP and glucose-6P (44). Carbon catabolite repression is poorly understood in F. tularensis; however, instances of catabolite repression have been described in other Gammaproteobacteria, including Pseudomonas aeruginosa and S. Typhimurium (45). Therefore, it is likely that F. tularensis also employs regulatory mechanisms to inhibit the utilization of alternative carbon substrates in the presence of a preferred carbon source such as glucose. We observed significant growth attenuation for a *pfkA* mutant in CDM supplemented with glucose relative to that in CDM alone or in CDM with glutamate. Similarly to that in E. coli, the buildup of glucose-6P may allosterically inhibit the activity of GlpX and prevent growth on gluconeogenic carbon sources, such as glutamate or other amino acids that are present at low concentrations in the medium.

Central carbon metabolism represents arguably the single most important cellular process in the context of bacterial viability and virulence. Energy generation, precursor biosynthesis, virulence factor expression, cell division, etc. are all contingent on a bacterium’s ability to acquire and utilize sufficient carbon to fuel these processes. Targeting bacterial catabolic and anabolic pathways is a promising strategy for combating pathogenic organisms such as F. tularensis. Indeed, it is well established that F. tularensis purine auxotrophs are attenuated during infection, and these mutants have been suggested as potential candidates for use as a live vaccine (46, 47). Similarly, targeting other essential metabolic pathways, such as gluconeogenesis, glycerol catabolism, or amino acid catabolism, either through drug or vaccine development, may constitute a means for limiting the spread of this deadly pathogen. Overall, by identifying the specific metabolic pathways and nutrients utilized by F. tularensis during intracellular growth, our findings begin to unravel the complex host-pathogen relationship exploited by F. tularensis during infection and further our understanding of F. tularensis pathogenicity.

## MATERIALS AND METHODS

### Bacterial and cell culture.

Francisella tularensis subsp. tularensis Schu S4 was obtained from BEI Resources and maintained in a biosafety level 3 (BSL-3) facility. Detailed protocols for bacterial and cell culture maintenance and manipulation are described in Text S1.

### Plasmid vectors and bacterial genetics.

Markerless, in-frame deletions were generated through allelic exchange, as previously described, for all F. tularensis deletion strains except for *glpA* (48). The *glpA* gene was disrupted using the Targetron system modified for use in Francisella species, as previously described (26). Detailed methods for mutant generation and complementation are provided in Text S1.

### Growth curves.

Overnight cultures of F. tularensis SchuS4 grown in CDM were diluted to an OD_600_ of 0.05 in 200 μl of CDM or modified CDM in a 96-well plate (Corning). Each major carbon source was added to a final concentration of 0.4%. Cultures were incubated in an Infinite 200M Pro series plate reader (Tecan) at 37°C with orbital shaking. Bacterial intracellular growth within J774A.1 or BMDM cells was determined by measuring the luminescence of Schu S4 harboring the luminescence reporter plasmid pJB2 or pJB3, described in Text S1. During infection, J774A.1 and BMDM cells were cultured in high-glucose (4.5 g/liter) or glucose-free, pyruvate-free DMEM (Gibco) supplemented with 10% dialyzed fetal bovine serum (FBS). When stated, BMDMs were pretreated 2 h prior to infection with 150 µM AICAR (Cayman Chemical) or Atglistatin (Cayman Chemical). Atglistatin cytotoxicity was measured using a Vybrant MTT cell proliferation assay kit (Thermo Fisher), following the manufacturer’s protocol. Detailed methods are provided in Text S1.

### Mouse infections.

Groups of 6- to 8-week-old female C57BL6/J mice (Jackson Labs) were inoculated intranasally with 100 CFU of F. tularensis Schu S4 WT or mutant strains. Infected and control mice were housed in a recirculating air system (Techniplast) within a BSL-3 facility. At 3 days postinfection, mice were sacrificed and the lungs, livers, and spleens were harvested and homogenized using a Biojector (Bioject). The homogenates were serially diluted and plated onto chocolate or MMH (modified Mueller Hinton) agar to quantify organ burdens.
