# Supplementary figures and images for: Defining the Metabolic Pathways and Host-Derived Carbon Substrates Required for Francisella tularensis Intracellular Growth
Source: mBio. 2018 Nov 20;9(6):e01471-18. doi: 10.1128/mBio.01471-18 (PMC6247087; doi:10.1128/mBio.01471-18)

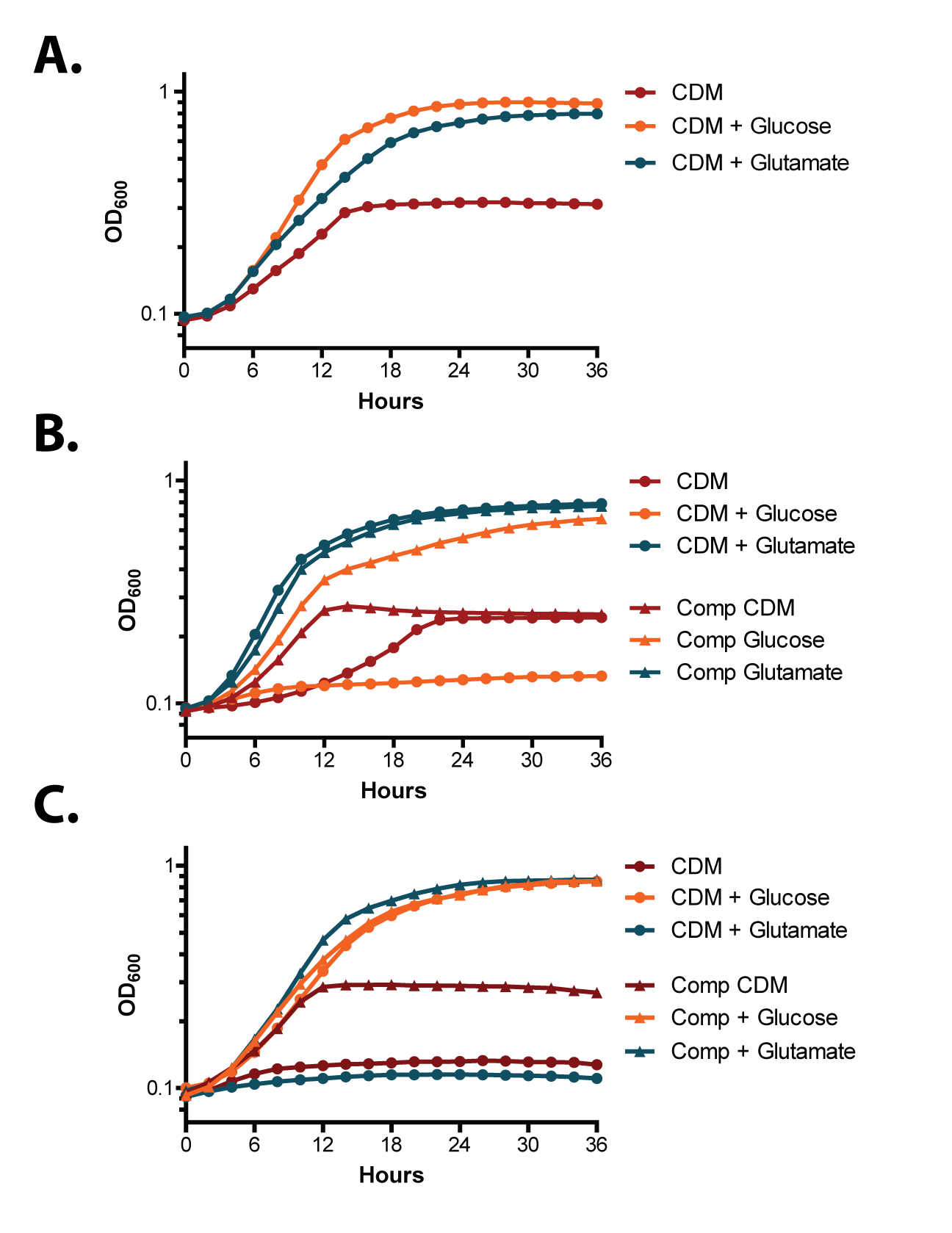

Supplement: FIG S1 [file mbo006184171sf1.tif]

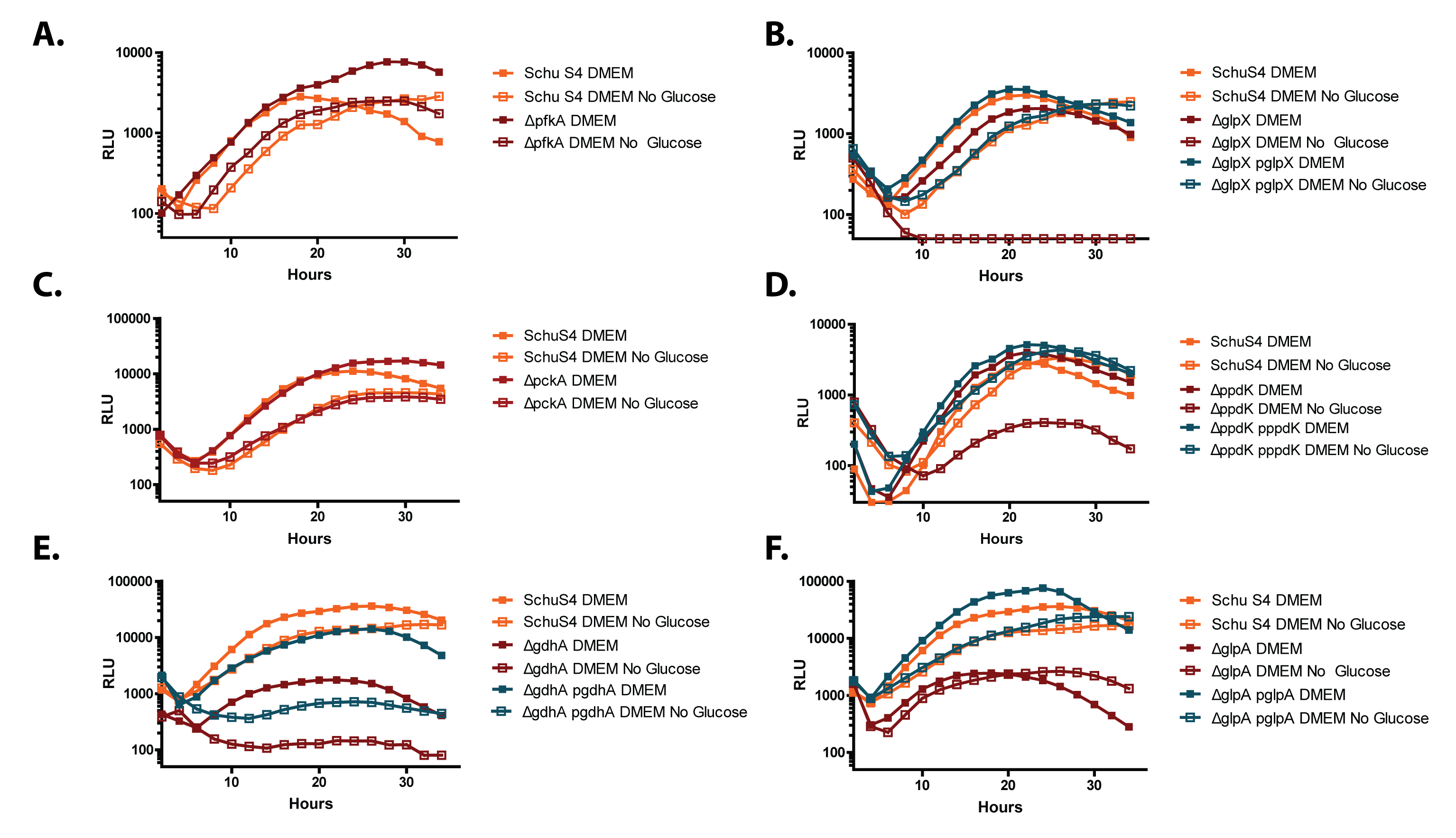

Supplement: FIG S2 [file mbo006184171sf2.tif]

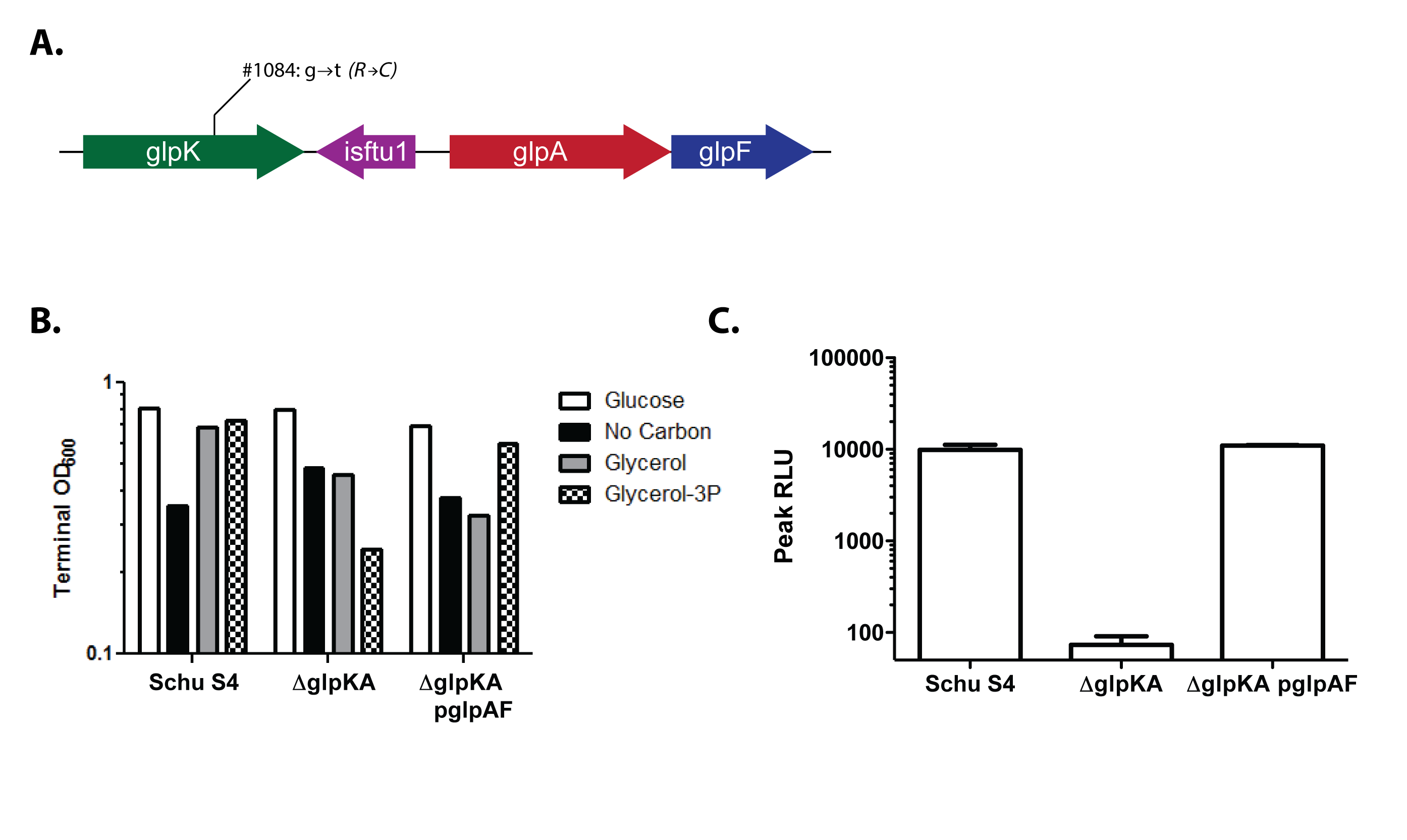

Supplement: FIG S3 [file mbo006184171sf3.tif]

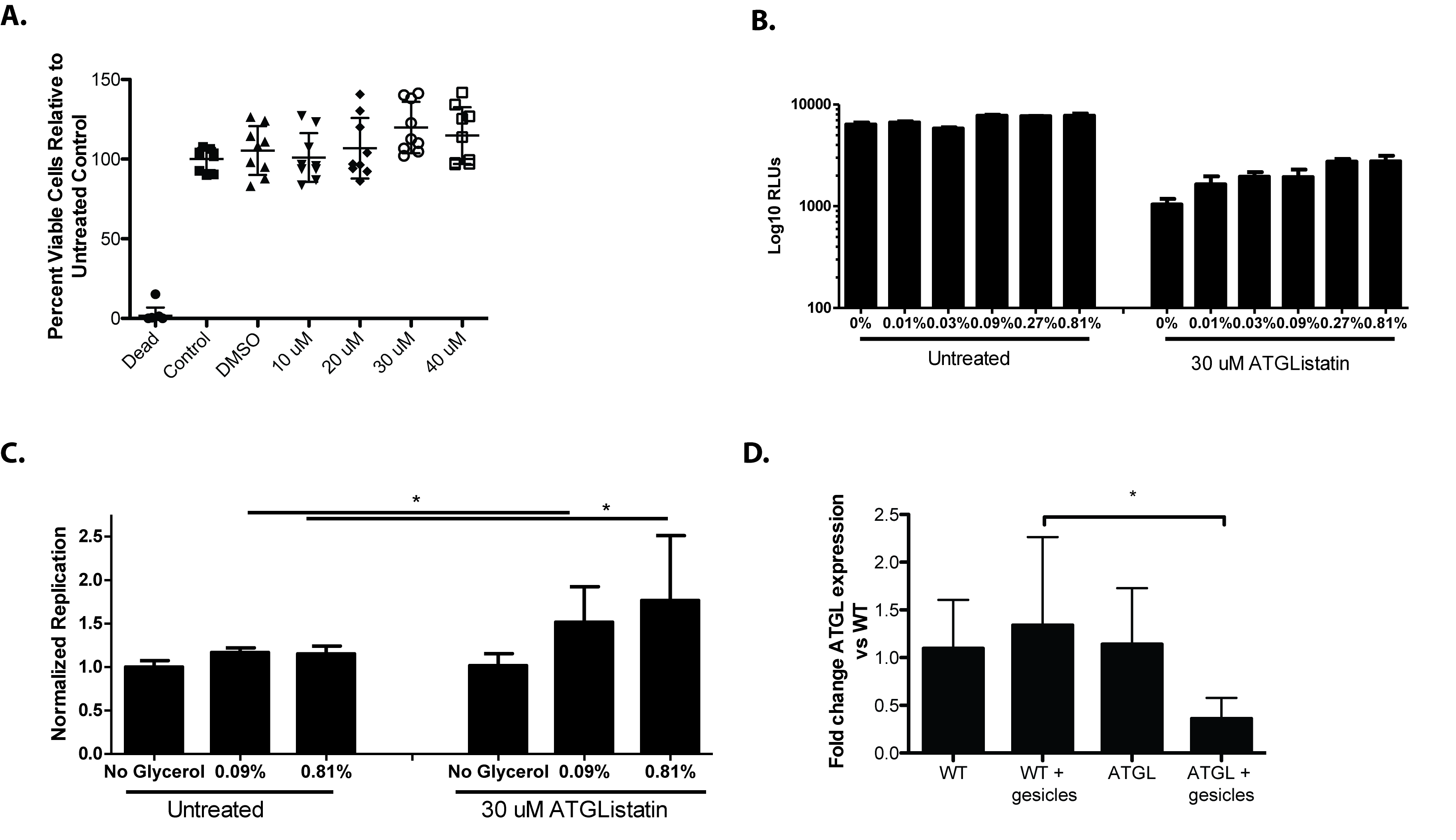

Supplement: FIG S4 [file mbo006184171sf4.tif]
