# Supplementary material for: Defining the Metabolic Pathways and Host-Derived Carbon Substrates Required for Francisella tularensis Intracellular Growth
Source: mBio. 2018 Nov 20;9(6):e01471-18. doi: 10.1128/mBio.01471-18 (PMC6247087; doi:10.1128/mBio.01471-18)
Supplement: TEXT S1 [file mbo006184171s1.docx]

**Supplemental Materials and Methods**

**Bacterial Strains**

*Francisella tularensis* subspecies *tularensis* Schu S4 was obtained from BEI Resources. *F. tularensis* was maintained on solid chocolate agar medium supplemented with 1% IsoVitaleX (Becton-Dickson); modified Mueller-Hinton (MMH) agar supplemented with 1% tryptone, 0.5% NaCl, 0.05% L-cysteine freebase, 1% glucose and 0.00025% Fe pyrophosphate; brain heart infusion (BHI) broth supplemented with 1% IsoVitaleX; Chamerlains Defined Media (CDM); or modified CDM^1^. For selection, each growth medium was supplemented with 10μg/ml kanamycin or 200 μg/ml hygromycin when applicable. All cloning was performed in *Escherichia coli* DH5α and S17-1λpir strains propagated in Luria-Bertani (LB) broth or solid agar supplemented with 50μg/ml kanamycin or 200μg/ml hygromycin when necessary for selection*.* All cultures were grown at 37°C with aeration.

**Cell Culture**

J774A.1 (ATCC TIB-67) macrophage-like cells were maintained in Dulbecco’s modified Eagle medium (DMEM) supplemented with 4.5 g/liter glucose, 10% fetal bovine serum, 2mM L-glutamine, and 1mM sodium pyruvate. Bone marrow derived macrophages (BMDMs) were generated from C57BL6 mice (Jackson Labs) by flushing bone marrow cells from murine femurs and incubating the recovered cells for 6 days in L929 cell-conditioned DMEM containing 10% fetal bovine serum. Prior to use, non-adherent cells were removed by washing BMDMs with phosphate-buffered saline (PBS), and cells were recovered from untreated plates using 10mM EDTA in PBS. For experiments, BMDMs were maintained in high glucose (4.5g/L) DMEM supplemented with 2mM L-glutamine and 10% fetal bovine serum.

**Generating ATGL-Deficient BMDMs**

Bone marrow from 8-14 week old C57Bl/6J or ATGL-flox (B6N.129S-Pnpla2^tm1Eek^/J, Jackson Labs Stock No. 24278) were generated as described above. 5 days after the bone marrow is cultured, 20 ul of Cre Recombinase Gesicles (Takara) were added directly to the culture media on the developing cells. Approximately 36 hours later, the cells were harvested and incubated overnight in tissue culture dishes for experiments. Almost all BMDMs took up detectable levels of the Cre Recombinase Gesicles (data not shown). For the different replicate experiments, the effectiveness of this process on ATGL RNA in the ATGL-flox mice treated with gesicles was 46.7%, 21.5%, and 33.9% of C57Bl/6 mice not exposed to gesicles.

**qRT-PCR**

BMDMs were seeded at 250,000 cells the night before infection. Immediately prior to infection, Trizol (Life Technologies) was added to a subset of wells. The samples were treated with choloform and centrifuged. The top fraction was mixed with 70% ethanol and RNA was isolated using a RNEasy kit (Qiagen). Samples were then treated with DNase (ThermoFisher). The samples were analyzed using a Sensifast, One-Step PMaster mix kit (Bioline) following the manufacturer’s suggested ratio. The primers were based on experiments by Ogasawara *et al.* and verified for use in mice^2^. Briefly, The conditions for the PCR were: 10 min at 45 °C for reverse transcription, 2 min at 95 °C for polymerase activation, 35 cycles with a 95°C denature (10 sec), 55°C anneal (10 sec) and 72°C extension (30 sec). Wells that did not receive template or reverse transcriptase had no amplification in any replicate for any experiment. ATGL: 5′-AGTTCAACCTTCGCAATCTC-3′(sense), 5′-GTCACCCAATTTCCTCTTGG -3′(antisense). B-actin: 5′-ACCTGACAGACTACCTCATG-3′ (sense), 5-ACTCATCGTACTCCTGCTTG-3′ (antisense).

**Plasmid Vectors and Bacterial Genetics**

Markerless, in-frame deletions were generated through allelic exchange as described for all *F. tularensis* deletion strains except for *glpA*^3^*.* For allelic exchange, all suicide vectors were constructed from pEDL50, a modified version of the suicide vector pMP812 (Kan^r^, *sacB)* containing an origin of transfer for mating into *F. tularensis*^4^. The pEDL50 suicide vector was mated into *F. tularensis* Schu S4 via *E. coli* S17-1λpir by mixing the bacteria on LB agar overnight, then selecting for primary integrants on chocolate agar with kanamycin (10μg/mL) and polymyxin B (200μg/mL). Kan^r^, PMB^r^ resistant strains were grown overnight in BHI broth without selection to allow for recombination, then plated on chocolate agar containing 10% sucrose for counterselection (loss of plasmid). Deletion strains were confirmed through PCR and sequencing (Genewiz). The *glpA* gene was disrupted using the Targetron system modified for use in *Francisella* species. The Targetron suicide vector was created using primers assigned by the Targetron Primer design program (Sigma). The vector was transformed into *F. tularensis* Schu S4 and the mutant was isolated as described^5^. For complementation of deletion strains, selected genes and their predicted promoters were PCR amplified and ligated into pJB3, a luminescent reporter plasmid derived from the low-copy shuttle, pMP831 that constitutively expresses the *Photorhabdus luminescens luxCDABE* operon from pXB173^6^. Genes lacking an obvious native promoter were cloned into pJB2, a modified version of pJB3 that contains a *pblaB* promoter sequence driving expression of the targeted gene. Suicide and complementation vectors were transformed into *E.coli* S17-1λpir and *F. tularensis,* respectively*. E.coli* S17- λpir was transformed through heat shock. For *F. tularensis* transformation, Schu S4 was grown overnight in CDM, washed 4 times with 0.5M sucrose and electroporated in a 1mm gap cuvette at 2kV, 25μF, and 200Ω. The transformants were allowed to recover for 2 hours in BHI broth at 37°C, then plated on chocolate or MMH agar with appropriate selection.

**Growth Curves**

Overnight cultures of *F. tularensis* SchuS4 grown in CDM were diluted to an OD_600_ of 0.05 in 200μl of CDM or modified CDM in a 96-well plate (Corning). CDM base media (without glucose, glutamate, etc. as a primary carbon source) consisted of the following components:

| **Component** | **Final Concentration (mM)** |
| --- | --- |
| L-Aspartic Acid | 3 |
| DL-Isoleucine | 3 |
| L-Leucine | 3 |
| DL-Methionine | 2.7 |
| DL-Serine | 3.8 |
| L-Tyrosine | 2.2 |
| L-Cysteine | 1.7 |
| L-Arginine | 2.3 |
| L-Histidine | 1.3 |
| L-Lysine | 2.7 |
| L-Proline | 17.4 |
| DL-Threonine | 16.8 |
| DL-Valine | 3.4 |
| Calcium Pantothenate | 8.4 |
| Thiamine HCl | 11.9 |
| Spermine HCl | 19.8 |
| NaCl | 171.1 |
| MgSO4 7*H2O | 548.5 |
| FeSO4 7*H2O | 7.2 |
| KH2PO4 | 7.3 |
| K2HPO4 | 5.7 |

All CDM was buffered with 50mM MES at pH 6.2 to account for ammonia production during amino acid catabolism in experiments where modified CDM contained amino acids as the primary carbon source. Each major carbon source was added to a final concentration of 0.4%. Cultures were incubated in an Infinite 200M Pro series TECAN plate reader (TECAN) at 37°C with orbital shaking. The OD_600_ was measured every 15 minutes for 48 hours.

**Macrophage Infections**

Bacterial intracellular growth within J774A.1 or BMDM cells was determined by measuring the luminescence of Schu S4 harboring the luminescence reporter plasmid pJB2 or pJB3 described above. J774A.1 and BMDM cells were plated at 5x10^4^ cells per well in a 96-well white wall, white bottom polystyrene plate (Corning) the night before infection. Each well was inoculated at a multiplicity of infection (MOI) of 100. Following a two-hour infection period, the inoculation medium was removed and replaced with 200μl of media containing 25μg/ml (J774A.1) or 10μg/ml (BMDMs) gentamicin. Luminescence was measured every 15 minutes for 48 hours using an Infinite 200M Pro series TECAN plate reader (TECAN) maintaining constant 37°C temperature and 5% CO_2_. To enumerate intracellular bacteria by plating, BMDM tissue culture medium was removed 2 hours post-gentamicin treatment and cells were washed once with PBS before being scraped up, vortexed hard for 1 minute, diluted and plated on chocolate agar. When applicable, J774A.1 and BMDM cells were cultured in glucose-free, pyruvate-free DMEM (Gibco) supplemented with 10% dialyzed FBS. For the AICAR and Atglistatin experiments, BMDMs were pretreated 2 hours prior to infection with 150µM AICAR (Cayman Chemical), or Atglistain (Cayman Chemical), and this drug concentration was maintained throughout the infection. Atglistatin cytotoxicity was measured using a Vybrant MTT Cell Proliferation Assay Kit (ThermoFisher) following the manufacturer’s protocol.

**Mouse Infections**

Groups of 6-8 week old female C57BL6 mice (Jackson Labs) were inoculated intranasally with 100 CFU of *F. tularensis* Schu S4 wild-type or mutant strains. Infected and control mice were housed in a recirculating air Techniplast system (Techniplast) within a BSL-3 facility. At 3 days post infection, mice were sacrificed and the lungs, livers and spleens were harvested and homogenized using a Biojector (Bioject). The homogenates were serially diluted and plated onto chocolate or MMH agar to quantify organ burdens.

1. CHAMBERLAIN, R. E. EVALUATION OF LIVE TULAREMIA VACCINE PREPARED IN A CHEMICALLY DEFINED MEDIUM. *Appl. Microbiol.* **13,** 232–5 (1965).

2. Ogasawara, J., Sakurai, T., Kizaki, T., Ishibashi, Y., Izawa, T., Sumitani, Y., Ishida, H., Radak, Z., Haga, S. & Ohno, H. Higher Levels of ATGL Are Associated with Exercise-Induced Enhancement of Lipolysis in Rat Epididymal Adipocytes. *PLoS One* **7,** e40876 (2012).

3. LoVullo, E. D., Sherrill, L. A., Perez, L. L. & Pavelka, M. S. Genetic tools for highly pathogenic Francisella tularensis subsp. tularensis. *Microbiology* **152,** 3425–3435 (2006).

4. LoVullo, E. D., Molins-Schneekloth, C. R., Schweizer, H. P. & Pavelka, M. S. Single-copy chromosomal integration systems for Francisella tularensis. *Microbiology* **155,** 1152–63 (2009).

5. Rodriguez, S. A., Davis, G. & Klose, K. E. Targeted gene disruption in Francisella tularensis by group II introns. *Methods* **49,** 270–4 (2009).

6. Brunton, J., Steele, S., Miller, C., Lovullo, E., Taft-Benz, S. & Kawula, T. Identifying Francisella tularensis genes required for growth in host cells. *Infect. Immun.* **83,** 3015–25 (2015).
