# Supplementary material for: Defining the Metabolic Pathways and Host-Derived Carbon Substrates Required for Francisella tularensis Intracellular Growth
Source: mBio. 2018 Nov 20;9(6):e01471-18. doi: 10.1128/mBio.01471-18 (PMC6247087; doi:10.1128/mBio.01471-18)
Supplement: TABLE S1 [file mbo006184171st1.docx]

| **TABLE S1** Growth of Schu S4 and *glpKA* strains in broth, BMDMs or J774A.1 cells | | | | | | |
| --- | --- | --- | --- | --- | --- | --- |
|  | CDM | CDM +  Glucose | CDM +  Glycerol | CDM +  G3P | BMDM | J774A.1 |
| WT | + | ++ | ++ | ++ | ++ | ++ |
| *ΔglpKA* | + | ++ | - | - | - | + |
| *ΔglpKA* p*glpAF* | + | ++ | - | ++ | ++ | + |
| *ΔglpKA* p*glpKAF* | + | ++ | ++ | ++ | ++ | + |
