# Supplementary material for: Defining the Metabolic Pathways and Host-Derived Carbon Substrates Required for Francisella tularensis Intracellular Growth
Source: mBio. 2018 Nov 20;9(6):e01471-18. doi: 10.1128/mBio.01471-18 (PMC6247087; doi:10.1128/mBio.01471-18)
Supplement: TABLE S2 [file mbo006184171st2.docx]

| **TABLE S2.** Summary table of Schu S4 WT and mutant strain growth in described infection models | | | | | | | |  |
| --- | --- | --- | --- | --- | --- | --- | --- | --- |
|  | CDM | CDM +  Glucose | CDM +  Glutamate | CDM +  Glycerol-3P | BMDM | J774A.1 | C57BL6/J | |
| WT | + | ++ | ++ | ++ | ++ | ++ | ++ | |
| *ΔpfkA* | + | - | ++ | *n/a* | ++ | ++ | ++ | |
| *ΔglpX* | - | ++ | - | *n/a* | - | - | - | |
| *ΔpckA* | + | ++ | ++ | *n/a* | ++ | ++ | + | |
| *ΔppdK* | - | ++ | - | *n/a* | ++ | + | ++ | |
| *ΔgdhA* | - | ++ | - | *n/a* | - | - | + | |
| *ΔglpKA* | + | ++ | *n/a* | - | - | + | - | |
